# Determination of *Giardia duodenalis* assemblages and multi-locus genotypes in patients with sporadic giardiasis from England

**DOI:** 10.1186/s13071-015-1059-z

**Published:** 2015-09-04

**Authors:** Corrado Minetti, Kenneth Lamden, Caroline Durband, John Cheesbrough, Andrew Fox, Jonathan M. Wastling

**Affiliations:** Institute of Infection and Global Health, University of Liverpool, Liverpool, UK; Public Health England Centre Cumbria and Lancashire, Chorley, UK; Lancashire Teaching Hospitals NHS Foundation Trust, Preston, UK; Public Health England Food Water and Environmental Microbiology Laboratory, Preston, UK; Faculty of Natural Sciences, Keele University, Keele, UK

**Keywords:** Giardiasis, *Giardia duodenalis*, Assemblage, Genotyping, Multi-locus genotype, MLG, Molecular epidemiology, United Kingdom

## Abstract

**Background:**

The protozoan *Giardia duodenalis* is a common but highly diverse human parasite that comprises a complex of seven morphologically identical genetic assemblages, further divided into sub-assemblages. There is very little information available on the diversity of *Giardia* sub-assemblages and multi-locus genotypes infecting people in the United Kingdom. In this study we studied the molecular epidemiology of *Giardia* in symptomatic patients from North West England.

**Methods:**

Whole faecal DNA was extracted from the faecal samples of 406 *Giardia* cases and the parasites assemblage, sub-assemblage and multi-locus genotype were determined using PCR amplification, DNA sequencing and phylogenetic analysis of the beta-giardin, glutamate dehydrogenase, triose-phosphate isomerase and small-subunit ribosomal RNA genes. Information about age, gender and self-reported clinical outcomes was also collected from the patients to check for differences associated with the infecting *Giardia* assemblage.

**Results:**

Our results showed a difference in the age prevalence of the two assemblages, with assemblage A being more common in older cases. Cases infected with assemblage B more often reported vomiting and a longer illness than cases infected with assemblage A. The majority of infections (64 %) were caused by assemblage B followed by assemblage A (33 %), while mixed-assemblage infections were rare (3 %). Assemblage A isolates mostly belonged to the sub-assemblage AII and showed completed identity with previously described isolates. The level of genetic sub-structuring was significantly higher in assemblage B isolates, since a higher proportion of novel assemblage B sequences was detected compared to assemblage A. A high number of assemblage B sequences showed heterogeneous nucleotide positions that prevented the unambiguous assignment to a specific sub-assemblage. Both previously described and novel multi-locus genotypes were described in both assemblages, and up to 17 different assemblage B multi-locus genotypes were found.

**Conclusions:**

We have produced the first data on the parasite multi-locus genotypes in the UK and have demonstrated that the molecular diversity of *Giardia* is similar to other developed countries. Furthermore, we showed that the parasite assemblages infecting humans may be associated with patients of different ages and with different clinical outcomes.

**Electronic supplementary material:**

The online version of this article (doi:10.1186/s13071-015-1059-z) contains supplementary material, which is available to authorized users.

## Background

Giardiasis is one of the most common parasitic gastrointestinal diseases in humans worldwide, and it is considered a neglected tropical disease [1]. It is caused by the parasitic protozoan *Giardia duodenalis*, which is transmitted either through contaminated water and food or from person to person.

This parasite is a highly diverse organism and a complex of at least seven morphologically identical genetic assemblages (named A to G) that can infect a variety of mammalian hosts [2]. The parasite assemblages are currently distinguished by polymerase chain reaction (PCR) and DNA sequencing of genes such as the small-subunit ribosomal RNA (*ssu-rRNA*), β-giardin (*bg*), glutamate dehydrogenase (*gdh*) and triose-phosphate isomerase (*tpi*) genes. There is increasing phenotypic and molecular evidence that these assemblages actually represent distinct species, and a new nomenclature using species names has been advocated [3]. Humans are infected by parasites belonging to assemblage A (*G. duodenalis*) and B (*G. enterica*), and the latter is usually the most prevalent in human populations [4].

There is extensive genetic sub-structuring within assemblage A and B, and parasites are further differentiated by sequence data from multiple loci into at least five so-called sub-assemblages (named AI-III and BIII-IV) [2]. A multi-locus sequence typing (MLST) approach and nomenclature based on the use of three genes (*bg*, *gdh* and *tpi*) has been proposed for assemblage A isolates [5]. However, assemblage B parasites show a considerably higher genetic variability and their unambiguous classification in sub-assemblages and sub-types is partially hampered by the frequent occurrence of DNA sequences with overlapping nucleotides (e.g. the presence of two possible nucleotides at one particular position in the sequence).

So far only a few studies have applied the MLST protocol to genotype *Giardia* from humans [5–7], and more data are needed on the distribution and diversity of multi-locus genotypes of *Giardia* in developed countries.

In particular, very little information is available on the assemblage and sub-assemblage diversity of *Giardia* in people from the United Kingdom. Previous studies have included only a relatively small number of patients, and have relied on the use of a single locus for parasite genotyping. Using restriction fragment length polymorphism analysis of the *tpi* gene assemblage B was found in 63.6 % of 33 *Giardia* patients from London, followed by assemblage A (27.3 %) and mixed-assemblage infections (9.1 %) [8]. Another study on symptomatic cases in London found a prevalence of 24, 73 and 3 % for *Giardia* assemblage A, B and mixed infections respectively by DNA sequencing of the *tpi* and *ssu-rRNA* genes [9]. A very similar picture has been reported more recently using a real-time *tpi* PCR assay on specimens from patients in England and Wales: assemblage B accounted for 72 % of the infections whereas mixed infections were very rare (3 %) [10]. Only two studies determined the *Giardia* sub-assemblage based on the sequencing of the *tpi* locus [8, 9]: all assemblage A parasites were AII, whereas both BIII and BIV sub-assemblages were identified in assemblage B isolates.

No study from the UK so far has investigated the molecular diversity of *Giardia* parasites in a large number of patients using a multi-locus sequence typing approach. Here we report the first data on the prevalence and diversity of *Giardia* assemblages, sub-assemblages and multi-locus genotypes in symptomatic patients with giardiasis from North West England, determined using the MLST protocol based on the sequencing of the *bg*, *gdh* and *tpi* loci.

## Methods

### Faecal specimen collection and DNA extraction

Faecal specimens were collected between January 2008 and August 2013 from patients with symptomatic giardiasis diagnosed by the microbiology laboratories of three hospitals in North West England: the Royal Preston Hospital serving the population of Central Lancashire), the Royal Blackburn Hospital serving East Lancashire and the Manchester Royal Infirmary serving the (Greater Manchester area. In all three laboratories all the faecal samples from diarrhoeic patients submitted by general practitioners are routinely tested for *Giardia*. Diagnosis at all laboratories was made using first an antigen detection method for the simultaneous detection of *Giardia* and *Cryptosporidium* (*GIARDIA/CRYPTOSPORIDIUM CHEK*®, Techlab) followed by confirmation of *Giardia* infection with an immunochromatographic assay (RIDA®QUICK *Giardia*, R-Biopharm). Specimens were also examined for *Cryptosporidium*, *Campylobacter, Salmonella*, *Shigella*, *Escherichia coli* O157 and *Vibrio* spp. when indicated, and patients found to be co-infected with one of these pathogens were excluded. Information about the patients’ gender, age, experienced symptoms and history of travel was collected via a questionnaire. Faecal specimens were stored unpreserved at 4 °C before DNA extraction. Due to logistical constraints samples collected from 2008 to 2010 were extracted in 2011 whereas samples collected in 2011 were extracted regularly within one month from collection. Whole faecal DNA was extracted directly from the specimens using a commercial kit (QIAamp® DNA Stool Mini Kit, QIAGEN Ltd.) following the manufacturer’s instructions with only minor modifications: in the first lysis step the samples were heated for 10 min at 95 °C (instead of 5 min at 70 °C as per the manufacturer’s instructions). The DNA was eluted in 100 μl of elution buffer and stored at −20 °C.

### *Giardia* multi-locus genotyping and phylogenetic analysis

Extracted DNA was used to amplify the *Giardia* beta-giardin (*bg*), glutamate dehydrogenase (*gdh*) and triose-phosphate isomerase (*tpi*) genes. For the *bg* gene, the 753 bp fragment of the gene was first amplified using the primers G7 (5′-AAGCCCGACGACCTCACCCGCAGTGC-3′) and G759 (5′-GAGGCCGCCCTGGATCTTCGAGACGAC-3′) [11], from which the 511 bp fragment was then amplified with the BGf (5′-GAACGAACGAGATCGAGGTCCG-3′) and BGr (5′-CTCGACGAGCTTCGTGTT-3′) [12]. For the *gdh* gene, a 754 bp fragment of the gene was first amplified using the primers GDH1 (5′- TTCCGTRTYCAGTACAACTC-3′) and GDH2 (5′-ACCTCGTTCTGRGTGGCGCA-3′), followed by the amplification of a 530 bp fragment with the GDH3 (5′- ATGACYGAGCTYCAGAGGCACGT-3′) and GDH4 (5′- GTGGCGCARGGCATGATGCA-3′) primers [5]. Two *Giardia* assemblage-specific nested PCR assays were used to amplify the *tpi* gene. A 605 bp fragment of the gene was first amplified using the AL3543 (5′-AAATIATGCCTGCTCGTCG-3′) and AL3546 (5′-CAAACCTTITCCGCAAACC-3′) primers [13]. Then the PCR products were used in two separate reactions, one involving the amplification of a 332 bp fragment of the gene from assemblage A using the Af (5′- CGCCGTACACCTGTCA-3′) and Ar (5′- AGCAATGACAACCTCCTTCC-3′) primers [14], and the other amplifying a 400 bp fragment from assemblage B with the primers Bf (5′- GTTGTTGTTGCTCCCTCCTTT-3′) and Br (5′-CCGGCTCATAGGCAATTACA-3′) [6]. In order to ensure the assemblage typing of most of the isolates, samples that failed to amplify at the *bg*, *gdh* or *tpi* loci were further amplified for the 292 bp fragment of the *ssu-rRNA* gene using the primers RH11 (5′-CATCCGGTCGATCCTGCC-3′) and RH4 (5′- AGTCGAACCCTGATTCTCCGCCAGG-3′) [15].

All PCR reactions were prepared in a final volume of 25 μl containing 2.5 units of *Taq* DNA polymerase and 1× buffer (containing Tris-Cl, KCl, (NH_4_)_2_SO_4_ and 15 mM MgCl_2,_ pH 8.7) (QIAGEN^®^), 200 μM of each deoxynucleotide (Sigma-Aldrich^®^), 250 nM of each primer, nuclease-free water and genomic DNA (2 μl in first step reactions and 1 μl of first reaction product in the nested reactions). Positive (genomic DNA from *G. duodenalis* assemblage A isolate WBC6 trophozoites and from an assemblage B confirmed clinical isolate) and negative (nuclease-free water) control samples were included in each reaction. Reactions were performed using a DNA Engine Dyad^®^ Peltier Thermal Cycler (MJ Research Inc.). All PCR reactions included a first denaturation step at 94 °C for 3 min, followed by 35 cycles each including denaturation at 94 °C for 20 s, annealing for either 30 s (*bg* gene: 65 and 64 °C for the primary and nested reaction, respectively; *gdh* gene: 58 and 68 °C for the primary and nested reaction, respectively; *ssu-rRNA* gene: 59 °C) or 45 s (*tpi* gene: 58, 68 and 62 °C for the primary reaction and the assemblage A- and B-specific secondary reactions, respectively) and extension at 72 °C for 1 min. All reactions were concluded by a final extension step at 72 °C for 10 min. Successful amplification was verified by running 5 μl of PCR products onto a 1.5 % agarose gel stained with SYBR^®^ Safe DNA gel stain (Invitrogen™). PCR products were purified using the QIAquick® PCR Purification kit (QIAGEN Ltd.), and they were sequenced in both directions using the respective forward and reverse primers with an Applied Biosystems^®^ 3730 DNA Analyser at the Core Genomic Facility, Medical School, University of Sheffield, UK. The chromatograms were analysed and the sequences edited using BioEdit (version 7.0.9). Sequences were then compared to homologous sequences (>99 % similarity) in GenBank using the Basic Local Alignment Search Tool (BLAST) (http://blast.ncbi.nlm.nih.gov/Blast.cgi) to determine the *Giardia* assemblage, sub-assemblage and sub-type. Maximum likelihood (ML) phylogenetic trees were built using the Molecular Evolutionary Genetics Analysis (MEGA) program ver. 6.06 (Tamura *et al.*, 2013) after determining the optimal nucleotide substitution model. Sequences without overlapping nucleotide positions were aligned in MEGA with representative sequences from the major *G. duodenalis* sub-assemblages AI-III and BIII-IV downloaded from GenBank. Also representative sequences from the animal assemblages (C-G) and other species of *Giardia* (either *G. muris* or *G. ardeae* depending on the availability of a sequence of the gene examined) were included. The full list of the reference sequences used in the phylogenetic analysis is reported in the Additional file 1.

### Statistical analysis

Statistical analysis was performed using SPSS^®^ Statistics 20 (IBM, USA). Tests of significance included the Pearson’s Chi-Square test (or Fisher’s Exact test for sparse data) and the Mann-Whitney’s U test(s) for categorical and continuous variables, respectively. Statistical significance was set as a *p*-value <5 %.

### Ethics, consent and permissions

Ethical approval for the use of the patients faecal samples and questionnaire information was obtained through the National Research Ethics Service, Committee North West, Lancaster under the following two names: Molecular typing of human *Giardia* isolates v3.2(REC reference 10/H1015/79) and Case–control study of risk factors for giardiasis in North West England (REC reference 11/NW/0704).

## Results

### PCR amplification success and assemblages prevalence

During the study period a total of 406 faecal specimens from patients with confirmed giardiasis were collected and extracted, and 218 (54 %) successfully amplified at least one of the three MLST genes tested. The *tpi* assemblage A/B-specific product amplified in 188 of these samples (86.2 %), followed by the *bg* gene in 166 (76.1 %) and the *gdh* gene in 153 (70.2 %). Of the 188 negative specimens that were further amplified for the *ssu-rRNA* gene, 77 (41 %) amplified for this locus whereas 111 (59 %) were negative. By considering the amplification of the four loci tested, 295 samples out of the 406 extracted (73 %) successfully amplified at least one of them. The overall PCR amplification success of the samples varied accordingly to the year of collection and the time from collection to DNA extraction. Specimens collected in 2012 and 2013 that were extracted within one month showed a higher amplification success rate (169 positives/199 tested, 85 %) compared with specimens from 2011 extracted between two and eight months (41/57, 72 %) and specimens from 2008 to 2010 that were stored unpreserved at 4 °C for more than one year before extraction (85/150, 57 %).

The *Giardia* assemblage was successfully determined from 247 specimens by either DNA sequencing (239 specimens) or on-gel visualization of the *tpi* assemblage-specific products (eight specimens for which DNA sequencing failed): assemblage B was found in the majority of the specimens (64 %, *n* = 158), followed by assemblage A (33 %, *n* = 82) and mixed-assemblage infections (3 %, *n* = 7). Of the seven specimens showing a mixed infection three were diagnosed by the *tpi* assemblage-specific PCR (whereas they were typed as B at both the *bg* and *gdh* loci), three were typed as B at the *tpi* locus but as A at the *gdh* locus (two specimens) or at the *bg* locus (one specimen), and one was typed as A at the *bg* locus but as B at the *gdh* locus.

### Socio-demographics, clinical and travel history of assemblage A and B patients

**Fig. 1:**
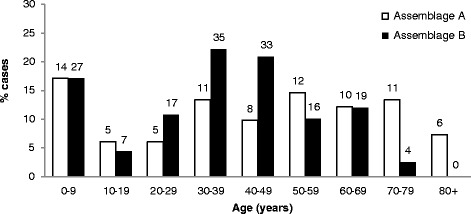
Infecting *Giardia* assemblage and age in 240 case-patients successfully genotyped and with a single-assemblage infection. The raw number of patients is reported above the bars

The two assemblages did not differ in their gender distribution (Pearson’s χ^2^, *p* = 0.494), but assemblage A cases had a significantly higher age (48.5 years, range 11 months-94) compared to B cases (38 years, range 1–79) (Mann-Whitney’s U test, *p* = 0.007): this was due to an excess of assemblage B infections in adults from 20 to 50 years of age compared with an excess of assemblage A infections in the elderly (70+ years) (Fig. 1).

The experienced symptoms and travel history of assemblage A and B patients are shown in Table 1. Patients with assemblage B infection reported vomiting significantly more often and longer illness duration compared with patients with assemblage A infection, whereas the opposite was seen for the presence of blood in stools. Although the frequency of overseas travel among patients with the two assemblages was similar, the majority of patients who visited countries more at-risk for giardiasis (e.g. Asia and Middle East, Africa and Latin America) were infected with assemblage B whereas those visiting countries not at-risk were mostly infected with assemblage A. Destinations reported by the 21 assemblage B patients included mostly India and Pakistan (47.6 %, ten patients), followed by South-East Asia (14.3 %, three) and Egypt (9.5 %, two).

**Table 1:** Clinical and travel history of patients with *Giardia* assemblage A or B infection

			Assemblage n (%)**		
Variable		No.*	A	B	*p*-value
Diarrhoea		141	49 (96.1)	89 (98.9)	0.296
Blood in stools		129	5 (11.1)	1 (1.2)	0.019
Vomiting		130	18 (40)	52 (61.2)	0.027
Abdominal pain (cramps)		135	34 (71)	69 (79)	0.268
Fever		126	19 (45.2)	40 (48)	0.801
Median length of illness in days (range), in patients no longer ill when contacted		72	10 (1–50)	18 (4–73)	0.019
Admitted to hospital due to illness		133	8 (16.3)	5 (6)	0.070
Travel abroad (outside UK)		142	16 (31.4)	23 (25.3)	0.435
Travel abroad to at-risk destinations	No travel abroad	142	35 (68.6)	68 (74.7)	<0.001
	Abroad, not at-risk destination		11 (21.6)***	2 (2.2)***	
	Abroad, at-risk destination~		5 (9.8)***	21 (23.1)***	

*number of participants that answered the question; **number of A and B patients with percentages calculated relative to those that answered the question; ~ including Asia and Middle East (with Turkey), Africa, Central and South America; ***proportions differing significantly from each other with *p* < 0.05

### *Giardia* sub-assemblages and subtypes at the *bg*, *gdh* and *tpi* loci

The phylogenetic trees based on the successfully sequenced and unambiguously identified *bg*, *gdh* and *tpi* loci are shown in the Additional file 2. No intra-assemblage variation was observed in the 34 samples that were sequenced at the *ssu-rDNA* locus so these results are not presented. A list for the sequences that showed novel nucleotide substitutions or overlapping nucleotides at any positions are shown in Additional file 3.

#### Assemblage A

At the *bg* locus, the majority of 42 isolates successfully and unambiguously identified showed complete sequence identity with the two previously described sub-assemblage AII subtypes A2 (16 isolates) and A3 (23), whereas one isolate had a sequence identical to the sub-assemblage AI reference. Two isolates (13/C146, 10/H12) showed a novel nucleotide substitution compared with the AII and AI references respectively, and three isolates showed overlapping nucleotides along the *bg* sequence.

At the *gdh* locus, of the 49 assemblage A isolates successfully identified the sequence of 27 isolates was identical to the sub-assemblage AII reference, whereas 18 and three isolates matched the two previously described sub-assemblage AII human isolates ECUST2196 and ISSGd198, respectively. The sequence of one isolate (12/C41) showed a novel substitution compared with the ECUST2196 isolate sequence, and two other isolates showed overlapping nucleotides at one position along the *gdh* sequence.

At the *tpi* locus, 50 successfully identified assemblage A isolates showed complete sequence identity with the sequence of the sub-assemblage AII reference, whereas one isolate showed a sequence identical to a previously described AI-type dog isolate (Martha). No *tpi* sequences with novel substitutions or overlapping nucleotides at any position were observed.

#### Assemblage B

At the *bg* locus, 16 different subtypes were identified in 89 unambiguously identified assemblage B. The majority (70 isolates) had sequences identical to those of five previously reported subtypes named B1 from humans: the most represented subtype was B1–3 (48 isolates), followed by B1–2 (11), B1–5 (six), B1–1 (three) and B1 (one). The sequence of seven isolates showed complete identity with the BIII reference sequence. One isolate was identical to the sub-assemblage BIV reference sequence, and two isolates showed complete identity with two previously described BIV-type isolates (Sweh198 and GU417). The sequences of six isolates were identical to those of three previously described subtypes from humans, namely BG-Ber2 (four), BG-Ber1 and BG-Ber6 (one each). Four isolates showed novel nucleotide substitutions in the *bg* sequence and 18 had overlapping nucleotides at multiple positions along the sequence.

At the *gdh* locus, 13 different subtypes were observed amongst the 69 isolates unambiguously identified. The majority (45 isolates) had a sequence that was identical to the reference BIV subtype. The sequence of 13 and two isolates matched the sequence of two previously described human (ISSGd167) and primate (Ad-158) isolates, respectively. Nine isolates showed novel substitutions (from two up to eight) in their *gdh* sequence, and 25 had overlapping nucleotides at multiple positions along the sequence.

In 86 isolates successfully sequenced and identified at the *tpi* locus, 13 different subtypes were found. The majority (50 isolates) had a sequence identical to the reference BIV, followed by 13 isolates where the sequence was identical to the sequence of the previously described BIV-type human isolate VB906855. A total of eleven isolates were identical to the sub-assemblage BIII reference subtype. Three isolates were identical to three BIII–type human isolates previously described (HS29, Sweh060 and HS114) and a further two isolates showed complete sequence identity with another previously described human isolate (Sweh171). Six isolates showed novel substitutions in their *tpi* sequence, and 24 had overlapping nucleotides at multiple positions along the sequence.

### *Giardia* multi-locus genotypes (MLGs) and household clusters

In total, 30 assemblage A and 45 assemblage B isolates were successfully and unambiguously genotyped at all the three MLST loci (Table 2). All assemblage A MLGs belonged to the sub-assemblage AII. Two previously identified MLGs (AII-1 and AII-2) were found in nine and four isolates respectively, and also four novel MLGs were identified. Three isolates genotyped at all the loci showed overlapping nucleotides in at least one sequence: one showed a potentially mixed AII-1/AII-2 MLG whereas the other two were not clearly identifiable. Regarding the assemblage B isolates, a higher diversity of MLGs was observed. With the exception of two previously identified MLGs (1 and 7) found in 21 (46.7 %) and one isolates, 15 novel assemblage B MLGs were identified showing different combinations of sequences at the three loci.

**Table 2:** *Giardia* multi-locus genotypes of 76 isolates unambiguously genotyped at the *bg*, *gdh* and *tpi* loci

Assemblage	No. isolates (ID)	*Bg*	*Gdh*	*Tpi*	MLG
A	9	A2	AII	AII	AII-1^a^
	4	A3	AII	AII	AII-2^a^
	9 (9/10, 11/29, 11/67, 12/C65, 12/C70, 12/C74, 13/C117, 13/C122, 13/C162)	A3	ECUST2196	AII	AII new (a)
	5 (8/H3, 11/27, 12/C16, 12/G, 13/C123)	A2	ECUST2196	AII	AII new (b)
	2 (12/C31, 13/C164)	A3	ISSGd198	AII	AII new (c)
	12/C41	A2	12/C41~	AII	AII new (d)
B	21	B1–3	BIV	BIV	1^b^
	1	B1–1	BIV	BIV	7^b^
	3 (12/C7, 13/C147, 13/CF)	B1–2	BIV	BIV	B new (a)
	3 (12/C87, 12/C92, 12/C96)	B1–5	BIV	VB906855	B new (b)
	3 (13/C152, 13/C165, 13/CE)	B1–5	ISSGd167	VB906855	B new (c)
	2 (12/C9, 12/C84)	B1–2	BIV	VB906855	B new (d)
	2 (12/C80, 12/C160)	B1–2	ISSGd167	BIV	B new (e)
	12/C95	B1–1	ISSGd167	BIV	B new (f)
	13/CO	13/CO~	BIV	BIV	B new (g)
	13/C149	B1–2	Ad-158	Sweh171	B new (h)
	13/C145	B1–2	Ad-158	VB906855	B new (i)
	13/C155	B1–3	13/C155	BIV	B new (l)
	12/C83	B1–3	ISSGd167	BIV	B new (m)
	11/28	B1	ISSGd167	BIV	B new (n)
	10/28	BIII	10/28~	VB906855	B new (o)
	12/C20	BIII	BIV	BIV	B new (p)
	12/C19	12/C19~	12/C19~	BIII	B new (q)

The isolates showing a novel MLG are specified by their identifier code (ID). ~ new sequence; ^a^same MLG reported by [5]; ^b^same MLG reported by [7]

Of the 13 patients that were part of five distinct household clusters identified, 12 had the infecting *Giardia* assemblage successfully determined. Assemblage A was found in two clusters. In one cluster where two family members were successfully genotyped the *Giardia* MLG causing infection was the same. Assemblage B was found in three clusters. In a cluster involving four people from whom the parasite MLG was determined the same MLG was found in all the cases.

## Discussion

We have characterized for the first time the molecular diversity of *Giardia* from a large number of symptomatic patients in North West England and we have found important differences in the age distribution and clinical outcome of infection between the parasite assemblage A and B.

The two assemblages differed in their age distribution in our sample with a higher prevalence of assemblage B in adults in their 30s and 40s and a predominance of assemblage A in older people. The difference between the two assemblages, in particular, was almost entirely concentrated in patients 70 years old or older, which showed an excess of assemblage A occurrence. The prevalence of *Giardia* assemblages in relation to the age of the patients has not been investigated thoroughly before, so we cannot compare directly our results with those from other studies. Although both assemblages showed a bimodal age distribution in a previous study in the UK [9], assemblage B was more prevalent than A in children and adults in their 30s, whereas the opposite was seen in people in their forties and particularly in the elderly (60+ years of age). These results were similar to what we have observed in our sample. The presence of age-related differences in the exposure to a particular assemblage or immunity to a particular parasite type could both be responsible for the observed pattern, but more studies are needed to test these hypotheses.

We also found differences in the self-reported clinical outcomes of infection between cases infected with the two assemblages. Vomiting was reported more frequently by the cases infected with assemblage B, who also reported a longer duration of illness compared to cases infected with assemblage A. Interestingly, there were no differences in relation to the reporting of other symptoms such as abdominal pain or fever. Whether these symptoms represent a general feature of infection due to similarities between the assemblages in term of host-parasite interactions, is an issue that needs further investigation. Overall our results suggest that infection with assemblage B may be associated with a more severe illness. In children from Cuba assemblage B was associated with a higher frequency of diarrhoea, flatulence and abdominal pain [16], and an association between assemblage B infection and flatulence was also observed in children from Sweden [7]. Only symptomatic cases of infection were included in our study. If assemblage B causes a more severe illness in humans, people infected with this assemblage will attend their general practitioner more frequently and they will then represent the majority of notified cases. This may explain the higher prevalence of assemblage B infections compared to those caused by assemblage A observed in symptomatic patients from the UK and other developed countries [2, 4]. More data are needed to determine whether assemblage A commonly occurs in people that are asymptomatic or show a relatively mild symptomatology in developed countries like the UK, as this has been reported in a few occasions from other places like Ethiopia [17], Saudi Arabia [18] and Cuba [16].

Interestingly, hospitalisation and the presence of blood in the stools were reported more frequently by assemblage A cases. This finding was probably due to an effect of age: overall assemblage A cases were older than B cases, and cases reporting blood in stools and hospitalisation were also older than those not reporting these two outcomes.

Another interesting finding was that the majority of cases with a history of travel to developing countries were infected with assemblage B. To the best of our knowledge, this is a new finding and the reasons behind such patterns require further investigation. It may be possible that due to the fact that *Giardia* assemblage B is considered to have a mostly human reservoir, the exposure to environments that are heavily contaminated with human faeces as in developing countries settings may lead to a higher proportion of infections with this particular assemblage in travellers.

The combined use of four different loci allowed the successful PCR amplification in more than 70 % of the extracted specimens. In particular, the use of the *ssu-rRNA* locus ensured the amplification in specimens that were negative at the *bg*, *gdh* and *tpi* loci. The small-subunit ribosomal RNA gene usually shows a high success of PCR amplification due to its multi-copy nature, particularly in specimens (like faeces or environmental samples) with a low quantity of target DNA or a higher quantity of PCR inhibitors [19].

The differential amplification success we observed at the MLST loci has been commonly reported in studies using the same primers, although results vary. The amplification success rate reported by a study on symptomatic patients from Germany was 92.4 % for the *bg*, 44 % for the *tpi* and only 25.7 % for the *gdh* locus [20]. Failure of amplification of assemblage B at multiple loci including those used in our study was also extensively observed in dog samples [21]. It has been proposed that the lack of amplification of particular loci could be due to the presence of nucleotide mismatches between the PCR primers and the genomic sequences [20], leading to the inability of certain primers to amplify particular sub-genotypes as suggested for the *bg* primers in respect to particular assemblage B sub-types [22]. We checked for the presence of PCR inhibition by spiking a subset of negative samples with *Giardia* DNA and re-amplifying them for the *ssu-rRNA* and *bg* loci, and they all successfully amplified. It has to be noted that our specimens originated from patients in whom *Giardia* infection was only confirmed using the commercially available enzyme-linked immunoassay method *GIARDIA/ CRYPTOSPORIDIUM CHEK*^*®*^. It has been shown that this assay detects the presence of a soluble antigen that is released in large quantities during the encystation of *Giardia* trophozoites *in vitro* [23]. It may be possible that in some of the specimens only the soluble antigen but not parasite cysts were present, resulting in the absence of *Giardia* DNA available for PCR amplification. Also the time passed from sample collection to DNA extraction negatively influenced the amplification success in the specimens, particularly those that were stored unpreserved for several months or even years before being extracted. This effect was likely due to the degradation of cysts and parasite DNA over time.

In terms of the prevalence and molecular diversity of the parasites, we found that assemblage B was responsible for the majority of infections. Our findings match those reported from symptomatic patients from Europe [6, 7, 20] and confirmed the prevalence data that has been previously published from the UK [8–10].

The molecular diversity of assemblage A isolates at the sub-assemblage level was successfully characterised in the majority of cases. Sequences with heterogeneous positions were observed only in a small proportion of isolates (and they were mostly observed at the *bg* locus), confirming the rare occurrence of sequences with overlapping nucleotides consistently reported in assemblage A parasites [4]. Regardless of the gene analysed, the vast majority of the sequences matched those of previously described isolates and sequences showing novel polymorphisms were observed in very few cases. Most of the assemblage A isolates were assigned to the sub-assemblage AII consistently across the three loci, confirming the preponderance of this sub-assemblage in humans compared to sub-assemblage AI as previously shown in other studies using multi-locus sequence typing [5–7].

The degree of variability (e.g. the number of different sub-types identified) differed between the loci and showed remarkable similarities with previous studies. At the *bg* locus the subtype A3 was found in the majority of isolates, as reported from symptomatic patients in Belgium [6] and Germany [20]. Two subtypes were found at the *gdh* locus, with AII being the most frequent as observed in both Belgium [6] Sweden [7], whereas only one AII subtype was identified at the *tpi* locus. The multi-locus analysis revealed that several patients were infected with two previously described sub-assemblage AII MLGs (AII-1 and AII-2), which have been previously reported in symptomatic patients from Italy [5], Belgium [6] and Sweden [7]. As reported in the aforementioned studies, we also observed MLGs showing different combinations of *bg*, *gdh* and *tpi* subtypes that have never been reported before. These results suggest that humans can be infected with an array of very diverse assemblage A genotypes, and that the MLST approach should be consistently used in all future molecular epidemiological studies in other geographical regions.

The molecular analysis of assemblage B isolates was complicated by the high frequency of occurrence of sequences with heterogeneous positions, with overlapping nucleotides observed on average in 20 % of the sequences across the three loci. A few assemblage B isolates showing heterogeneous templates potentially represented mixed sub-assemblage infections but the identification could not be unequivocally resolved. Heterogeneous sequences are a common occurrence in assemblage B parasites [4, 20], and their presence has been observed following DNA extraction and sequencing even from single *Giardia* cysts and between different cysts isolated from the same patient [24]. Other than mixed sub-assemblage infections, the occurrence of heterogeneous sequences may be due to the presence in *Giardia* of two nuclei, which are thought to accumulate mutations and evolve separately leading to allele sequence heterozygosity (e.g. the appearance of nucleotidic differences in the sequence between alleles of the same gene) [25]. The overall level of allele sequence heterozygosity has been estimated to be significantly higher in the genome of *Giardia* assemblage B compared with A [26], which can account for the higher occurrence of heterogeneous sequences observed in the former compared to the latter. The assignment to a particular sub-assemblage of isolates was not immediate also due to discrepancies between the different markers. At the *bg* locus the majority of the isolates belonged to subtypes that were part of the previously identified B1 group [6], as it has also been observed in human isolates from Sweden [7]. The B1 group clustered nearby the sub-assemblage BIII by phylogenetic analysis, but whether it represents an actual sub-assemblage other than BIII and BIV has to be verified. Conversely, the analysis of the *gdh* and *tpi* markers assigned the majority of isolates to the sub-assemblage BIV. The genotypic diversity observed in assemblage B isolates was much higher compared with the one observed within assemblage A, consisting in 21 novel sub-types (the majority of which at the *gdh* and *tpi* loci). Similar levels of diversity at the three markers were reported in assemblage B parasites in patients from Europe [7, 20]. There is still a lack of information about the level of genetic differentiation within *Giardia* assemblage B, and the classification in sub-assemblages can be complicated by the use of an imprecise terminology in naming parasite isolates [25], since the term sub-assemblage is commonly used to indicate nucleotidic variants that should be more appropriately referred to sub-types (within a sub-assemblage).

Interestingly, following multi-locus analysis of isolates successfully characterised at all the three markers we found the majority of patients being infected with one MLG previously reported in the majority of patients of a study in Sweden [7]. Similarly to what was observed for assemblage A, we also observed a high number of novel assemblage B MLGs showing different combinations of *bg*, *gdh* and *tpi* subtypes.

Our results confirmed the need for a novel DNA sequence-based classification system applicable for the genotyping of *Giardia* parasites below the assemblage level. Although the use of the *bg*, *gdh* and *tpi* loci seems to produce relatively consistent typing results for assemblage A sub-assemblages (supported by both phylogenetic and multi-locus analysis), it is clearly limited in its applicability for the assemblage B sub-typing. The extremely high sequence variation observed in assemblage B parasites (often involving a single nucleotidic difference between isolates) and the poor resolution of phylogenetic analyses complicate enormously the assignment of isolates to a specific sub-assemblage. The sub-groups BIII and BIV originally identified using allozyme electrophoresis are not supported by DNA sequence analysis [4]. The existence of several sub-assemblages within B (as suggested by the high levels of genetic variation) should then be re-evaluated following a more comprehensive biochemical and genetic characterization, in order to determine whether these subgroups are consistent and discrete. The use of next generation sequencing (NGS) technologies would be of great help in determining the extent of genetic sub-structuring within assemblages at a whole-genome level and in identifying either genes that are less affected by allele sequence heterozygosity or genes that are unique to particular sub-assemblages [25]. Such approach has been recently used to compare the genomes of two *Giardia* assemblage B isolates, leading to the identification of up to 70 polymorphic genes for potential use in sub-assemblage genotyping [27]. These genes could then be incorporated in multiplex real-time PCR assays allowing the sensitive and univocal identification of assemblage B sub-assemblages in faecal DNA samples.

## Conclusions

This study has shown that the molecular diversity of *Giardia* in symptomatic patients from North West England is largely similar to other areas of Europe, and that the two assemblages may differ in their distribution amongst people of different ages and in the illness they cause. The majority of infections were caused by assemblage B alone, followed by infections with sub-assemblage AII. Molecular analysis at both the single-locus and multi-locus level revealed the occurrence of both novel and already described sub-types, with assemblage B parasites showing a significantly higher diversity compared with assemblage A parasites. We have also generated the first data on the parasite multi-locus genotypes for the UK, showing the presence of both already described and novel genotypes. The unambiguous genotyping of a large number of assemblage B isolates was prevented by the high frequency of heterogeneous sequences. There is an urgent need for the development of novel and more robust genetic markers for this group of parasites to be incorporated into a reliable multi-locus genotyping method. The use of multi-locus sequence typing may help in the future to identify infection sources and the dynamics of spread in household clusters.

## Additional files

Additional file 1:
**Reference sequences used for** ***Giardia*** **sub-assemblage identification. Sequences are indicated by their GenBank accession number followed by the host of origin.** (XLSX 10 kb) Additional file 2:
**Maximum likelihood trees of 131** ***bg*** **(A), 118** ***gdh*** **(B) and 136** ***tpi*** **(C) sequences.** The trees are based on 442, 461 and 352 aligned positions of the *bg*, *gdh* and *tpi* sequences respectively. One representative sequence from each identified subtype is indicated by its study ID, and the total number of isolates sharing the same sequence is reported in parentheses. Reference sequences along with the matching previously described isolates (in red for assemblage A, in blue for assemblage B) are indicated by their GenBank accession number. Optimal nucleotide substitution models: Tamura-Nei with Gamma distribution (*bg*), Tamura 3 parameter with Gamma distribution (*gdh*), Kimura 2 parameter with Gamma distribution (*tpi*). Only bootstrap values ≥70 % for bipartitions are reported. (PDF 111 kb) Additional file 3:
**Sequences showing novel nucleotide substitutions or overlapping nucleotides in at least one position.** The isolate study ID is reported for each sequence. (XLSX 16 kb)
