# Supplementary figures and images for: Determination of Giardia duodenalis assemblages and multi-locus genotypes in patients with sporadic giardiasis from England
Source: Parasit Vectors. 2015 Sep 4;8:444. doi: 10.1186/s13071-015-1059-z (PMC4559006; doi:10.1186/s13071-015-1059-z)

(A) Bg

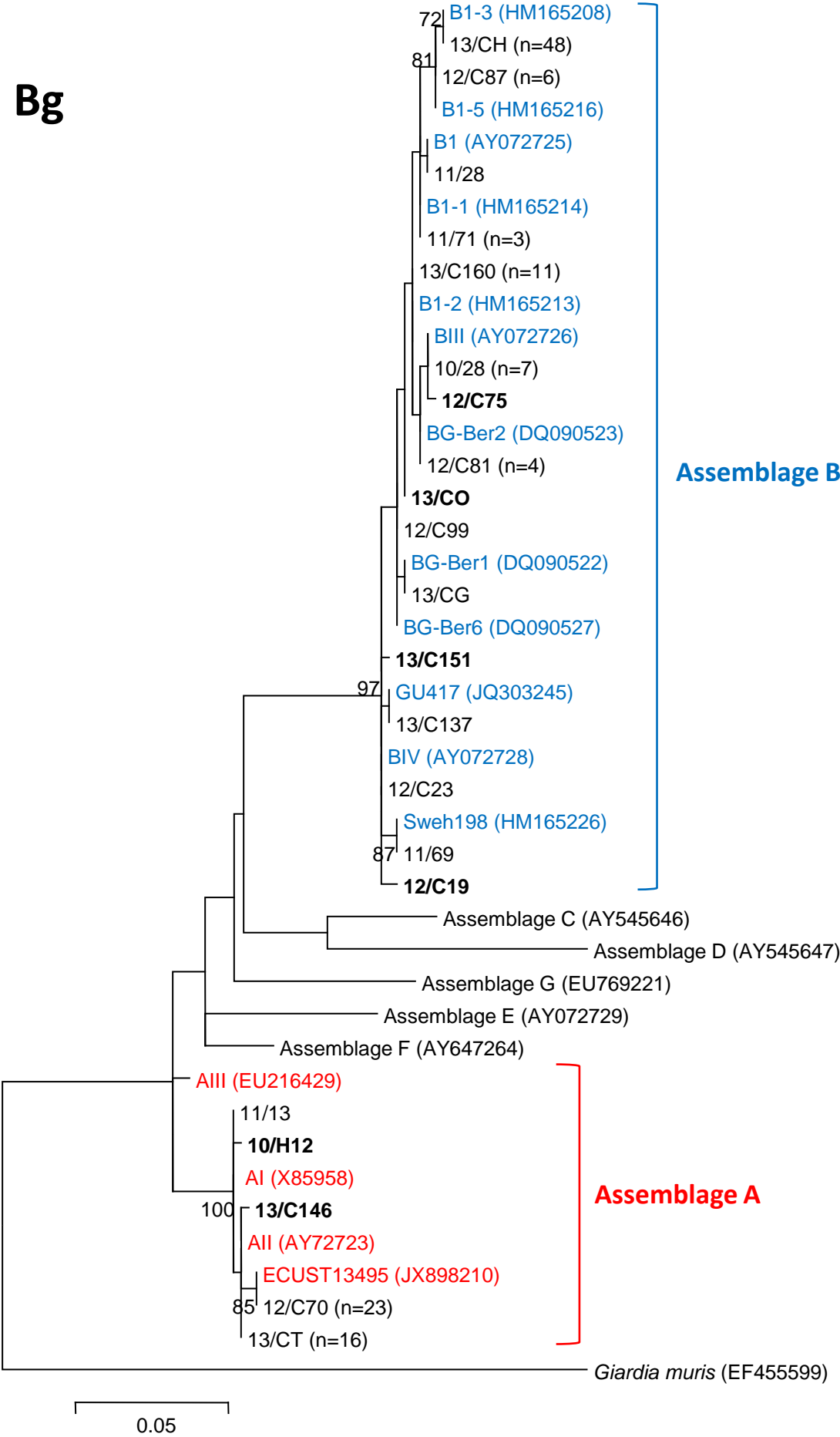

(B) Gdh

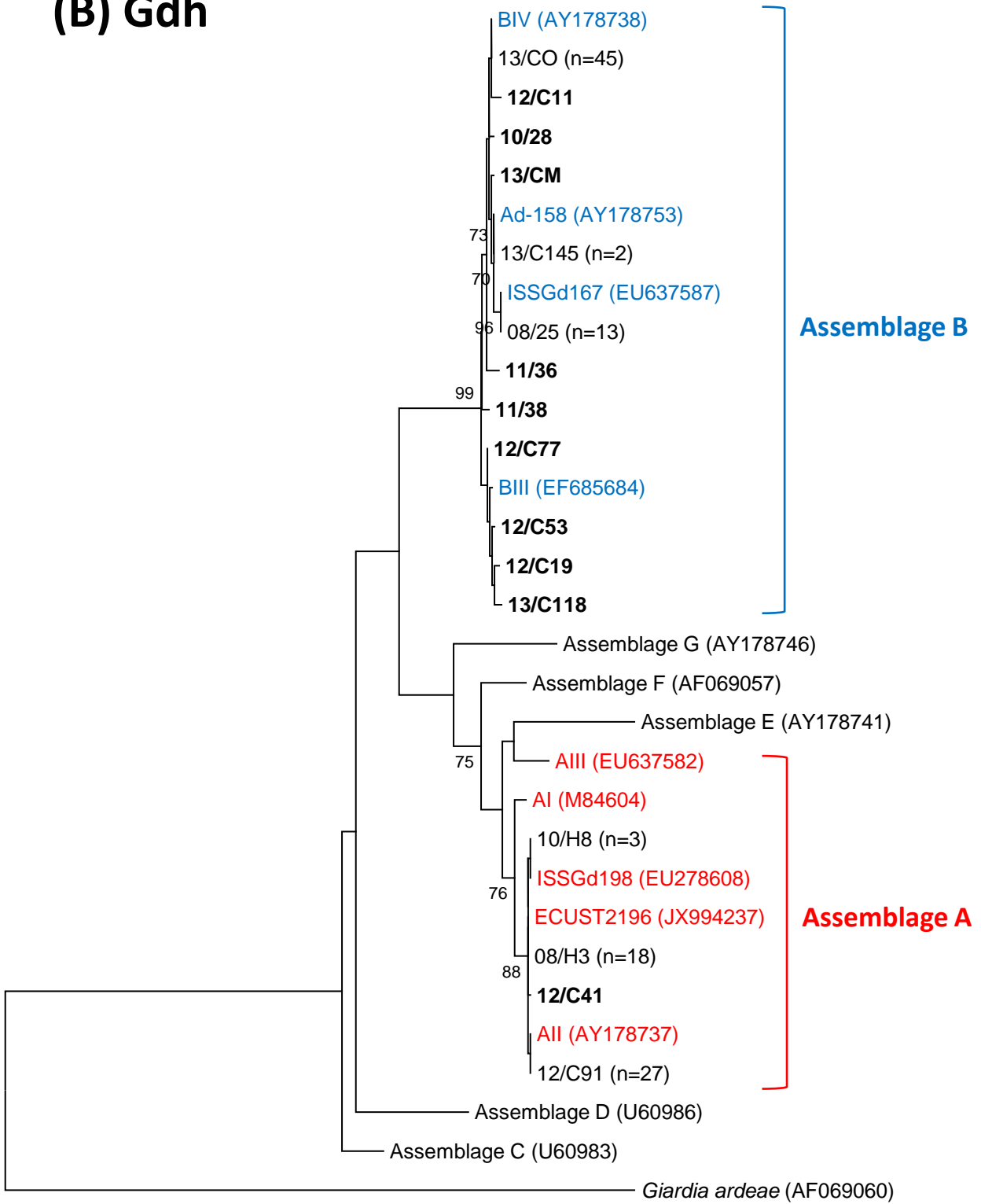

(C) Tpi

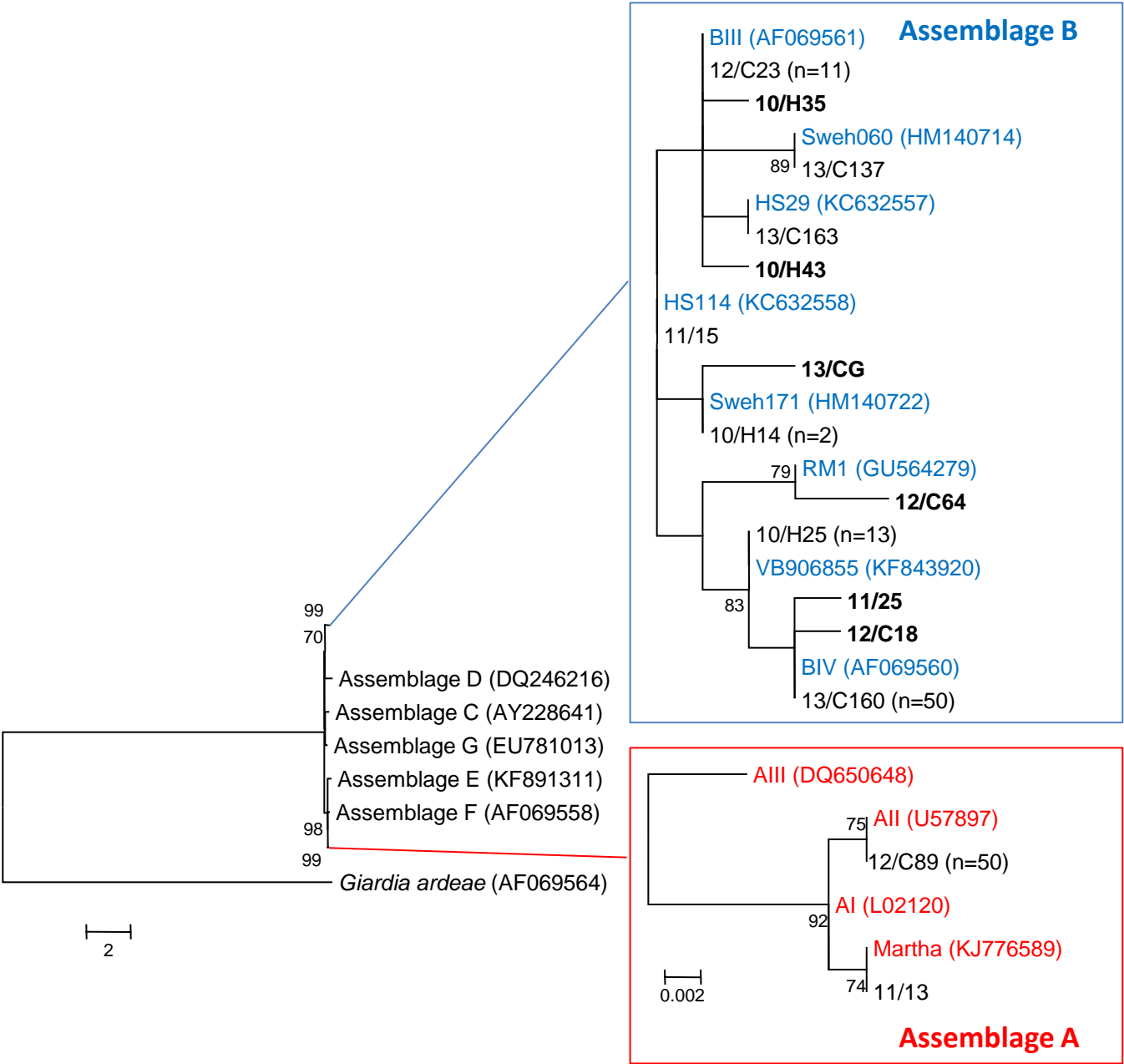

Supplement: Additional file 2: — Maximum likelihood trees of 131 bg (A), 118 gdh (B) and 136 tpi (C) sequences. The trees are based on 442, 461 and 352 aligned positions of the bg, gdh and tpi sequences respectively. One representative sequence from each identified subtype is indicated by its study ID, and the total number of isolates sharing the same sequence is reported in parentheses. Reference sequences along with the matching previously described isolates (in red for assemblage A, in blue for assemblage B) are indicated by their GenBank accession number. Optimal nucleotide substitution models: Tamura-Nei with Gamma distribution (bg), Tamura 3 parameter with Gamma distribution (gdh), Kimura 2 parameter with Gamma distribution (tpi). Only bootstrap values ≥70 % for bipartitions are reported. (PDF 111 kb) [file 13071_2015_1059_MOESM2_ESM.pdf]
